# The impact of generic substitution on the activities of pharmaceutical companies - a survey from the companies' perspective one year and five years after the introduction of generic substitution in finland

**DOI:** 10.1186/1472-6904-10-15

**Published:** 2010-10-22

**Authors:** Johanna Timonen, Marina Bengtström, Pekka Karttunen, Riitta Ahonen

**Affiliations:** 1 School of Pharmacy/Social Pharmacy, Faculty of Health Sciences, Kuopio Campus, University of Eastern Finland, P.O.Box 1627, FI-70211 Kuopio, Finland; 2 Pharma Industry Finland, Helsinki, Finland; 3 Siilinjärvi pharmacy, Siilinjärvi, Finland

## Abstract

**Background:**

Mandatory generic substitution (GS) was introduced in Finland on 1 April 2003. The aim of this study was to explore and compare the impacts of GS on the activities of pharmaceutical companies representing mainly original or generic pharmaceutical products in Finland. The self-reported impact of GS from pharmaceutical companies' perspective was explored with a focus on the number of employees, the range of sales packages on the market, the marketing activities, the research and development of new pharmaceutical products and storage of pharmaceuticals.

**Methods:**

A cross-sectional postal survey was conducted among pharmaceutical companies with an office in Finland and substitutable medicines in the Finnish pharmaceutical market one year (2004) and five years (2008) after the introduction of GS. Completed questionnaires were returned by 16 original and 7 generic product companies in 2004 (response rate 56%, n = 41) and by 16 original and 6 generic product companies in 2008 (response rate 56%, n = 39). Descriptive statistical analyses were performed.

**Results:**

The number of employees (2004: n = 6/16, 2008: n = 7/16) and the amount of prescription medicine marketing (2004: n = 7/16, 2008: n = 8/16) decreased in many of the original product companies after the introduction of GS. GS resulted in problems related to the storage of pharmaceuticals in the original product companies shortly after GS was introduced (p = 0.032 between 2004 and 2008). In the generic product companies, the prescription medicine representatives' visits to pharmacies increased at the beginning of GS (p = 0.021 between 2004 and 2008). In addition, GS caused problems with the storage of pharmaceuticals one year and five years after the reform (2004: n = 4/7, 2008: n = 3/6). The differences between original and generic product companies regarding the impacts of GS were not, however, statistically significant. GS did not affect on the range of sales packages on the market or the research activities of the majority of companies.

**Conclusions:**

The study suggests that GS has had impacts on the activities of pharmaceutical companies in Finland. There were also some differences, although not statistically significant, between the surveyed original and generic product companies regarding the self-reported impacts of GS. More investigations are needed in this field.

## Background

Increasing pharmaceutical expenditures have been a common problem in many western countries. One way to curb growth in pharmaceutical expenditures is through generic substitution (GS). GS has been introduced in at least 22 European countries [1-3] and in most states in the United States [4]. The purpose of GS is to increase dispensing of less expensive generic medicines and to decrease the price of pharmaceutical products through price competition. GS has successfully promoted the sales of less expensive medicines [5-7] and decreased the prices of pharmaceutical products [8-11] in several countries, and thus has effectively reduced growth in the pharmaceutical expenses of society and patients, for example in Sweden [12] and Finland [13].

GS has been a considerable pharmaceutical policy reform in many countries, not merely for society and patients, but also for parties involved in the pharmaceutical distribution chain (pharmaceutical companies, wholesale pharmaceutical companies and pharmacies). Even though GS has been introduced in many countries, there is a limited number of research publications in this area. Several studies have investigated pharmacists' opinions regarding GS and the implementation of GS in pharmacies in different countries [14-18]. Some studies have reported pharmacy owners' experiences of the impacts of GS on pharmacies in Denmark [15] and Finland [19]. The impact of GS on the activities of wholesale pharmaceutical companies [20], as well as the impact of GS on the sales of pharmaceutical companies [7] have been explored in Finland. However, there are no published studies concentrating on the impacts of GS on the activities, other than competition [8,10,11], of pharmaceutical companies.

Mandatory GS was introduced in Finland on 1 April 2003. Since then pharmacists have been obligated to substitute the least expensive or close to the least expensive medicine for a prescribed medicine if the prescribed medicine is not within the price corridor and the physician or the customer do not object to the substitution [21]. The price corridor is determined quarterly by the least expensive medicine in every substitution group, and it includes all the substitutable medicines with a price difference of less than €2 in products priced under €40, and of less than €3 in products priced €40 or more. Substitutable medicines are included on a list of interchangeable medicines compiled by the Finnish Medicines Agency [22]. Interchangeable medicines contain the same active ingredient in the same quantity, have the same pharmaceutical form (exception: tablets are substitutable for capsules and capsules for tablets), are bioequivalent and belong to a pharmaceutical group in which substitution can be performed safely. At the beginning of 2009, the list of interchangeable medicines comprised about 42% (n = 2 797) of the human pharmaceutical products with a marketing authorization in Finland (n = 6 694) [23].

Between 2003 and 2008, GS was possible for 12-24 million prescriptions reimbursed annually [21]. Customers objected to GS for 10-12% of dispensings and physicians objected for 0.2-0.4% of dispensings. The annual direct savings of the substitutions for patients and for society have been €26-36 million. GS also stimulated significant price competition between pharmaceutical companies [11], and estimated savings from the reduced price of medicines have been €34-59 million annually [13,21].

The pharmaceutical industry objected to GS before it was introduced [24,25]. According to the research-based pharmaceutical industry, promoting the use of generic products was desirable, but it should be achieved by allowing normal competitive mechanisms to function, not by mandatory GS [24]. In addition, it was argued that GS would have an impact on the pharmaceutical companies and their everyday activities, such as marketing [26] and the storage of pharmaceutical products [24]. It was also argued that GS would raise drug safety issues [24,26], decrease the range of different packages (e.g. strength, size, pharmaceutical form) of pharmaceutical products for sale, and reduce incentives for research and development of new pharmaceutical products in Finland [27].

The aim of this study was to explore and compare the impact of GS on the activities of pharmaceutical companies representing mainly original or generic pharmaceutical products from the companies' perspective one year and five years after the introduction of GS in Finland. The self-reported impact of GS was explored with a focus on the number of employees, marketing activities, the range of sales packages on the market, research and development (R&D) of new pharmaceutical products and storage of pharmaceuticals. Results reported in this article are part of a larger study exploring the impact of GS for pharmaceutical companies in Finland. Results concerning impact of GS on the turnover and gross margin of pharmaceutical companies have been published elsewhere [7].

## Methods

Two cross-sectional postal surveys were conducted one year (June-August 2004) and five years (November 2007-January 2008) after the introduction of GS. Questionnaires were sent to all pharmaceutical companies with an office in Finland and that market one or more substitutable medicines in Finland. The questionnaire was addressed to the key person in the company, such as the managing director or the sales and marketing director. The list of companies was obtained from the National Agency for Medicines' (NAM) list of the domestic holders of marketing authorization and Pharma Industry Finland's (PIF) list of member companies. The NAM list included pharmaceutical companies that have Finland as their home country in the NAM's register and that hold one or more valid marketing authorizations in Finland. PIF is a trade association for research-based pharmaceutical companies in Finland and most research-based pharmaceutical companies operating in Finland are members of PIF.

The Finnish pharmaceutical reference book (Pharmaca Fennica) on the Internet, and the current list of interchangeable medicines produced by NAM, were used to verify which companies marketed substitutable medicines in Finland. In 2008, the Medicinal product search service at the NAM's web site was also used, but it did not exist in 2004.

The lists of the NAM and PIF included 95 pharmaceutical companies or other parties operating in the Finnish pharmaceutical market in 2004. Of these, 43 pharmaceutical companies fulfilled the inclusion criteria (marketed substitutable medicines in the Finnish pharmaceutical market and had an office in Finland). The questionnaire was posted to all these 43 companies. After one reminder, 25 (58%) questionnaires were returned. However, two of the returned questionnaires were not included in the study because the company reported that they did not presently have substitutable medicines in the Finnish pharmaceutical market. Consequently, a total of 41 pharmaceutical companies (25 represented original products, 9 represented generic products and 7 represented both products) fulfilled the inclusion criteria, from which 23 (56%) companies returned completed and usable questionnaire.

In 2007-2008, the lists of the NAM and PIF contained 93 pharmaceutical companies or other parties operating in Finland. The questionnaire was sent to all 39 pharmaceutical companies (19 represented original products, 10 represented generic products, 7 represented both products and 3 represented parallel imported products) that marketed substitutable medicines in the Finnish pharmaceutical market and had an office in Finland. Of these companies, 29 were the same as in 2004. Three reminders were needed in the 2008 survey to achieve the same response rate than in 2004. After three reminders, 23 (59%) companies returned the questionnaire. However, one of the returned questionnaires was not complete and it was not included. Consequently, a total of 22 (56%) questionnaires were included in the final data analyses.

In 2004, the survey questionnaire included 37 structured or open-ended questions and one Likert-scale question. Responses to seven structured questions and two open-ended questions are reported in this article. In 2007-2008, the questionnaire included 27 questions, both structured and open-ended. The questions reported in this article were the same in 2004 and in 2007-2008, with the exception the minor changes described below. The questions related to the impact of GS on the number of employees, the amount of prescription medicine marketing, the prescription medicine representatives' visits to pharmacies and to physicians, the number of different packages of pharmaceutical products for sale, R&D of new pharmaceutical products and storage of pharmaceuticals. The questions were selected on the basis of debate about the possible impact of GS before it was introduced. The self-reported impact of GS on the number of employees, marketing activities, the range of packages for sale and R&D was elicited using similar structured questions. For example, the impacts of GS on the number of employees in the company were investigated with question, "Has generic substitution influenced the number of employees in your company?". The question had four response options: 1. No, 2. The number of employees has decreased, 3. The number of employees has increased, 4. Some other impact. In the fourth response option, the respondents were also requested to report what kind of impact. If the respondent answered that the number of employees had increased or decreased, they were asked to specify by how many employees. Respectively, if the respondent answered that the number of packages for sale had increased or decreased, they were asked to specify by the decrease or increase per cent in the number of packages. In 2004, an open-ended question was used to determine which department within each company was most impact on the number of employees by GS. In the 2007-2008 survey this information was sought using a structured question formulated using the results of the 2004 survey. The impact of GS on the storage of pharmaceuticals was investigated using a structured question with a list of seven options in 2004 and eight options in 2008, with respondents given the possibility to select several options. In all questions in 2004 and 2007-2008 surveys, the impact was assessed since the introduction of GS in Finland.

The data were analyzed with SPSS 14.0 statistical software. A descriptive approach was used in the analyses, using frequencies and cross-tabulations and Fisher's Exact test for a group comparison within the survey and between the surveys. Differences in the background characteristics between study companies in the 2004 and 2008 surveys were tested with Fisher's Exact tests and Mann-Whitney U tests. The significance level was defined as p-values ≤0.05.

## Results

### Background characteristics

Of the pharmaceutical companies that responded to the survey in 2004, 12 represented original products, 4 represented generic products and 7 represented both original and generic products. Of the companies that represented both products, 4 companies reported that they represent mainly original products and 3 companies reported that they represent mainly generic products. Of the companies that responded to the survey in 2008, 13 represented original products, 3 represented generic products and 6 represented both. Of the companies that represented both, 3 companies reported that they represent mainly original products and 3 companies reported that they represent mainly generic products. Because the number of study companies was small, the companies were categorized as companies that represented mainly original pharmaceutical products (original product companies) and companies that represented mainly generic pharmaceutical products (generic product companies). The background characteristics of the study companies in the 2004 and 2008 surveys are shown in Table 1. There were no statistically significant differences between the 2004 and 2008 survey groups of original product companies and generic product companies.

**Table 1:** Background characteristics of the study companies in the 2004 and 2008 surveys.

	Survey 2004 n of respondents (%)	Survey 2008 n of respondents (%)	P-value
**Number of companies**			1.000
All	23	22	
Original products	16 (70)	16 (73)	
Generic products	7 (30)	6 (27)	
**Number of employees**			
Original product companies			0.732
≤19	1 (7)	4 (25)	
20-49	1 (7)	2 (13)	
50-99	7 (50)	5 (31)	
100-149	2 (14)	2 (13)	
≥150	3 (22)	3 (19)	
Generic product companies			0.470
≤19	5 (71)	2 (40)	
20-49	-	-	
50-99	2 (29)	1 (20)	
100-149	-	1 (20)	
≥150	-	1 (20)	
**Turnover of the companies (€)**^a^			
Original product companies			0.381
≤1,000,000	-	-	
1,000,001-20,000,000	1 (7)	6 (37)	
20,000,001-40,000,000	5 (36)	2 (13)	
40,000,001-60,000,000	3 (22)	3 (19)	
60,000,001-80,000,000	2 (14)	1 (6)	
80,000,001-100,000,000	1 (7)	2 (13)	
≥100,000,001	2 (14)	2 (13)	
Generic product companies			0.755
≤1,000,000	1 (14)	1 (17)	
1,000,001-20,000,000	5 (72)	3 (50)	
20,000,001-40,000,000	1 (14)	-	
40,000,001-60,000,000	-	1 (17)	
60,000,001-80,000,000	-	-	
80,000,001-100,000,000	-	-	
≥100,000,001	-	1 (17)	
**Median proportion (%) of turnover coming from the sale of pharmaceutical products included on the list of interchangeable medicines**^b^			
All	25 (range 1.5-100)	11 (range 1-93)	0.749
Original product companies	23 (range 1.5-80)	8.5 (range 1-70)	0.527
Generic product companies	30 (range 5-100)	45 (range 5.4-93)	0.836
**Median proportion (%) of turnover coming from the sale of pharmaceutical products included in the price corridor**^b^			
All	10 (range 0-90)	8.2 (range 0-90)	0.767
Original product companies	7.5 (range 0-50)	3.5 (range 0-70)	0.608
Generic product companies	30 (range 5-90)	45 (range 5.4-90)	0.836
**Median market share (%) in the Finnish pharmaceutical market**^b^			
All	1.9 (range 0.1-10)	1.9 (range 0.1-9)	0.904
Original product companies	3.8 (range 0.6-10)	2.0 (range 0.3-8)	0.354
Generic product companies	0.3 (range 0.1-2)	0.3 (range 0.1-9)	0.662

^a^The companies reported their turnover from 2003 in the 2004 survey and from 2006 in the 2008 survey.

^b^The companies reported their proportion (%) from 2006 in the 2008 survey.

### Impact of GS on the number of employees

GS was reported to affect on the number of employees in most (2004: n = 9/16, 2008: n = 9/16) of the original product companies in both surveys and in most (n = 5/7) of the generic product companies in the 2004 survey (Table 2). In the original product companies the number of employees decreased (2004: n = 6, 2008: n = 7), and in the generic product companies the number of employees increased (2004: n = 3). In the 2008 survey, most (n = 4/6) of the generic product companies reported that GS had not impacted the number of employees, but the difference with the 2004 survey was not statistically significant (p = 0.283). In addition, the differences between the original and generic product companies were not statistically significant in the 2004 (p = 0.063) and 2008 (p = 0.814) surveys. Table 3 indicates that GS mainly affected the number of employees in the sales and marketing departments in the study companies.

**Table 2:** Impact of generic substitution on the number of employees in the pharmaceutical companies.

	Survey 2004				Survey 2008			
	Original product companies n (%)	Generic product companies n (%)	Total n (%)	P-value	Original product companies n (%)	Generic product companies n (%)	Total n (%)	P-value
Has generic substitution influenced the number of employees in your company?*			0.063					0.814
No	7 (44)	2 (29)	9 (39)		7 (44)	4 (67)	11 (50)	
Number of employees has decreased	6 (37)	2 (29)	8 (35)		7 (44)	2 (33)	9 (41)	
Number of employees has increased	-	3 (43)	3 (13)		2 (12)	-	2 (9)	
Some other impact^a^	3 (19)	-	3 (13)		-	-	-	
Total	16 (100)	7 (100)	23 (100)		16 (100)	6 (100)	22 (100)	

* P-value between the 2004 and 2008 surveys in the original product companies (p = 0.210) and in the generic product companies (p = 0.283)

^a ^Employees have been reassigned to another post, openings in the company have not been filled.

**Table 3:** Companies' departments most impacted on the number of employees by the introduction of generic substitution.

	Survey 2004	Survey 2008
	Pharmaceutical companies (n = 11)^a^ n (%)^b^	Pharmaceutical companies (n = 11) n(%)^b^
**Number of employees has decreased**		
Sales and marketing	6 (54)	8 (73)
Marketing authorization and reimbursement	-	1 (9)
Research and development	-	1 (9)
Logistics	-	-
Management	-	1 (9)
**Number of employees has increased**		
Sales and marketing	3 (27)	1 (9)
Marketing authorization and reimbursement	-	1 (9)
Research and development	-	-
Logistics	1 (9)	-
Management	-	-
**Some other impact**		
Sales and marketing	2 (18)	-

^a ^Two original product companies and one generic product company did not report the department, in which GS had influenced on the number of employees.

^b ^The companies could report several departments.

Of the original product companies with a decreased number of employees (2004: n = 6, 2008: n = 7), five in 2004 and four in 2008 reported a decrease in the number of employees since the introduction of GS. These companies reported a total decrease of 81 people (median 5, range 2-60) in the 2004 survey and 33 people (median 5.5, range 2-20) in the 2008 survey. In the 2008 survey, two of the original product companies reported an increase in number of employees, which was a total of five people.

For the generic product companies that reported increased number of employees in the 2004 survey (n = 3), the total increase was nine people (median 3, range 2-4) since the introduction of GS. For those companies that reported a decreased number of employees (n = 2) in the 2004 survey the total decrease was nine people. In the 2008 survey, only one of the generic product companies with a decreased number of employees gave the number of decreased employees, which was two employees.

### Impact of GS on the marketing of pharmaceutical products

Table 4 summarizes the impact of GS on the marketing activities of the study companies. In both surveys, more than half (2004: n = 9/16, 2008: n = 9/16) of the original product companies reported that GS had impacted the amount of prescription medicine marketing. Of these, most companies (2004: n = 7, 2008: n = 8) had decreased the amount of marketing after GS. In the generic product companies GS was reported to affect the prescription medicine representatives' visits to the pharmacies (n = 5/7) in the 2004 survey, but in the 2008 survey, most (n = 5/6) of the generic product companies reported that GS had not affected the representatives' visits to pharmacies (p = 0.021). The differences between original and generic product companies in relation to their marketing activities were not statistically significant in the 2004 and 2008 surveys (Table 4).

**Table 4:** Impact of generic substitution on the marketing of pharmaceutical products in the pharmaceutical companies.

	Survey 2004				Survey 2008			
	Original product companies n (%)	Generic product companies n (%)	Total n (%)	P-value	Original product companies n (%)	Generic product companies n (%)	Total n (%)	P-value
Has generic substitution influenced the amount of prescription medicine marketing?*				0.103				1.000
No	7 (44)	4 (57)	11 (48)		7 (44)	3 (50)	10 (45)	
Marketing has decreased	7 (44)	1 (14)	8 (35)		8 (50)	3 (50)	11 (50)	
Marketing has increased	-	2 (29)	2 (9)		1 (6)	-	1 (5)	
Some other impact^a^	2 (12)	-	2 (9)		-	-	-	
Total	16 (100)	7 (100)	23 (100)		16 (100)	6 (100)	22 (100)	
Has generic substitution influenced prescription medicine representatives' visits to pharmacies?**				0.120				0.619
No	9 (60)	2 (29)	11 (50)		10 (62)	5 (83)	15 (68)	
Visits have decreased	2 (13)	-	2 (9)		2 (13)	1 (17)	3 (14)	
Visits have increased	3 (20)	5 (71)	8 (36)		4 (25)	-	4 (18)	
Some other impact^b^	1 (7)	-	1 (5)		-	-	-	
Total	15 (100)	7 (100)	22 (100)		16 (100)	6 (100)	22 (100)	
Has generic substitution influenced prescription medicine representatives' visits to physicians?***				1.000				0.802
No	10 (63)	5 (71)	15 (65)		9 (60)	4 (67)	13 (62)	
Visits have decreased	5 (31)	2 (29)	7 (31)		5 (33)	1 (17)	6 (29)	
Visits have increased	-	-	-		1 (7)	1 (17)	2 (9)	
Some other impact^c^	1 (6)	-	1 (4)		-	-	-	
Total	16 (100)	7 (100)	23 (100)		15 (100)	6 (100)	21 (100)	

* P-value between the 2004 and 2008 surveys in the original product companies (p = 0.624) and in the generic product companies (p = 0.372)

** P-value between the 2004 and 2008 surveys in the original product companies (p = 1.000) and in the generic product companies (p = 0.021)

*** P-value between the 2004 and 2008 surveys in the original product companies (p = 1.000) and in the generic product companies (p = 1.000)

^a ^Marketing of pharmaceutical products within generic substitution has ended, marketing has been segmented and allocated.

^b ^The content of marketing has been changed.

^c ^Marketing has been focused on pharmaceutical products that do not include generic substitution and on new products.

### Impact of GS on the range of sales packages of pharmaceutical products on the market

Most of the original product companies in both surveys (2004: n = 8/15, 2008: n = 8/14) and most of the generic product companies in the 2004 survey (n = 4/7) reported that GS had not impacted the number of different sales packages on the market (Table 5). In the 2008 survey, two-thirds (n = 4/6) of the generic product companies reported that GS had influenced the number of different packages for sale, but the differences between the companies (p = 0.347) or surveys (p = 0.790) were not statistically significant.

**Table 5:** Impact of generic substitution on the range of sales packages on the market in the pharmaceutical companies.

	Survey 2004				Survey 2008			
	Original product companies n (%)	Generic product companies n (%)	Total n (%)	P-value	Original product companies n (%)	Generic product companies n (%)	Total n (%)	P-value
Has generic substitution influenced the number of different sales packages of pharmaceutical products on the pharmaceutical market in your company?*				0.318				0.347
No	8 (53)	4 (57)	12 (54)		8 (57)	2 (33)	10 (50)	
The number of packages has decreased	7 (47)	2 (29)	9 (41)		5 (36)	2 (33)	7 (35)	
The number of packages has increased	-	1 (14)	1 (5)		1 (7)	2 (33)	3 (15)	
Total	15 (100)	7 (100	22 (100)		14 (100)	6 (100)	20 (100)	

* P-value between the 2004 and 2008 surveys in the original product companies (p = 0.710) and in the generic product companies (p = 0.790)

Among the original product companies that reported a decrease in the number of sales packages on the market (2004: n = 7/15, 2008: n = 5/14) the median decrease in the number of packages was 10% (range 0.5-20%) in the 2004 survey. In the 2008 survey, three of the original product companies reported an average decrease of 15% (range 5-20%) of packages sold. Among the two generic product companies that reported a decrease in the number of packages for sale in the 2004 survey, the number of packages had decreased 3% and 10%. In the 2008 survey the generic product companies that reported a decreased number of sales packages (n = 2) did not report the percentage decrease.

### Impact of GS on the R&D of new pharmaceutical products in Finland

In both surveys, the majority of original (2004: n = 8/14, 2008: n = 8/12) and generic (2004: n = 2/3, 2008: n = 3/4) product companies that conducted R&D of new pharmaceutical products reported that GS had not influenced their R&D in Finland (Table 6).

**Table 6:** Impact of generic substitution on research and development of new pharmaceutical products^a^.

	Survey 2004				Survey 2008			
	Original product companies n (%)^a^	Generic product companies n (%)	Total n (%)^b^	P-value	Original product companies n (%)	Generic product companies n (%)	Total n (%)	P-value
Has generic substitution influenced the research and development of new pharmaceutical products in Finland in your company?*				0.161				0.154
No	8 (57)	2 (67)	10 (59)		8 (67)	3 (75)	11 (69)	
Research and development has decreased	5 (36)	-	5 (29)		4 (33)	-	4 (25)	
Research and development has increased	-	1 (33)	1 (6)		-	-	-	
Some other impact^c^	2 (14)	-	2 (12)		-	1 (25)	1 (6)	
Total	14 (107)	3 (100)	17 (106)		12 (100)	4 (100)	16 (100)	

* P-value between the 2004 and 2008 surveys in the original product companies (p = 0.594) and in the generic product companies (p = 1.000)

^a ^The table contains only the responses of companies that conducted research and development of new pharmaceutical products.

^b^One company chose two impacts (research and development has decreased and some other impact).

^c^For example, the research of pharmaceutical products within generic substitution does not interest no longer, research and development will be directed to new pharmacotherapy areas.

### Impact of GS on the storage of pharmaceutical products

GS was reported to affect the storage of pharmaceutical products in most of the original (n = 12/16) and generic (n = 4/7) product companies in the 2004 survey (Table 7). In the 2008 survey, most (n = 10/15) of the original product companies reported that GS had not impacted the storage of pharmaceutical products, and the difference compared to the responses of the original product companies in the 2004 survey was statistically significant (p = 0.032). Of the generic product companies, half (n = 3/6) reported that GS had impacted the storage in the 2008 survey. In the original product companies GS had most often caused over-stocking (2004: n = 9/16) and problems with the expiry dates (2004: n = 7/16). Problems with expiry dates (2004: n = 4/7, 2008: n = 3/6) and the availability (2008: n = 3/6) of pharmaceutical products were the most commonly reported problems by the generic product companies. The differences between original and generic product companies were not, however, statistically significant.

**Table 7:** Impact of generic substitution on the storage of pharmaceutical products by the pharmaceutical companies.

	Survey 2004				Survey 2008			
	Original product companies (n = 16) n (%)^a^	Generic product companies (n = 7) n (%)^a^	Total (n = 23) n (%)^a^	P-value	Original product companies (n = 15) n (%)^a^	Generic product companies (n = 6) n (%)^a^	Total (n = 21) n (%)^a^	P-value
*Has generic substitution influenced the storage of pharmaceutical products by your company?*								
No*	4 (25)	3 (43)	7 (30)	0.626	10 (67)	3 (50)	13 (62)	0.631
It has caused over-stocking**	9 (56)	2 (29)	11 (48)	0.371	4 (27)	1 (17)	5 (24)	1.000
It has caused problems with the expiry date of pharmaceutical products***	7 (44)	4 (57)	11 (48)	0.667	3 (20)	3 (50)	6 (29)	0.291
It has caused over-stocking of obligatory stocks^b ^****	4 (25)	1 (14)	5 (22)	1.000	3 (20)	-	3 (14)	0.526
It has caused problems with the availability of pharmaceutical products*****	4 (25)	1 (14)	5 (22)	1.000	2 (13)	3 (50)	5 (24)	0.115
It has complicated the execution of obligatory storage^b ^******	2 (12)	1 (14)	3 (13)	0.684	2 (13)	1 (17)	3 (14)	1.000
It has increased returns of pharmaceutical products from pharmacies after the new price corridor is determined^c^	-	-	-	-	2 (13)	1 (17)	3 (14)	1.000

* P-value between the 2004 and 2008 surveys in the original product companies (p = 0.032) and in the generic product companies (p = 1.000)

** P-value between the 2004 and 2008 surveys in the original product companies (p = 0.149) and in the generic product companies (p = 1.000)

*** P-value between the 2004 and 2008 surveys in the original product companies (p = 0.252) and in the generic product companies (p = 1.000)

**** P-value between the 2004 and 2008 surveys in the original product companies (p = 1.000) and in the generic product companies (p = 1.000)

***** P-value between the 2004 and 2008 surveys in the original product companies (p = 0.654) and in the generic product companies (p = 0.266)

****** P-value between the 2004 and 2008 surveys in the original product companies (p = 1.000) and in the generic product companies (p = 1.000)

^a^The companies could chose several impacts.

^b^In Finland, pharmaceutical companies were obligated to store enough pharmaceutical products in certain medicine groups (e.g. antibiotics, cardiovascular medicines, analgesics) to cover ten or five months of consumption of that product until the end of the year 2008. The obligatory stock of pharmaceutical products was based on the average sales of the product during the period from January to September of the previous year. The obligatory storage Act was amended in 2009.

^c^The question did not include this alternative in the 2004 survey.

## Discussion

In this study the impacts of GS on the activities of pharmaceutical companies representing mainly original or generic pharmaceutical products were investigated one year and five years after the introduction of GS in Finland. The original product companies self-reported that GS had impacted the number of employees and the amount of prescription medicine marketing one year and five years after the introduction of GS. In addition, GS caused problems related to the storage of pharmaceutical products shortly after it was introduced. According to the generic product companies, GS affected the prescription representatives' visit to pharmacies at the beginning of the reform. Furthermore, GS caused problems with the storage of pharmaceutical products one year and five years after the introduction. However, the differences between original and generic product companies regarding the impacts of GS were not statistically significant in this study. This might be because of the small study population.

After the introduction of GS the number of employees had decreased in many of the original product companies and, especially in the sales and marketing departments. The findings are in line with the statistics of PIF, where the number of employees increased steadily between 1990 and 2002 [28], but decreased after 2003 almost without exception [29-33]. In contrast at a European level the number of employees in the pharmaceutical industry was stable during the same period [34]. In Finland, the estimation of the overall decrease in employees is about 800-900 people between 2003 and 2008, due to the mergers of companies and downsizing e.g. especially in the marketing, but also in R&D [29-33]. In 2008, the total number of employees in the pharmaceutical industry was about 6 000 people. GS stimulated competition in the Finnish pharmaceutical market [11,35]. Reducing the number of employees was one response to this new competition between the companies in Finland [7]. However, the decrease in the number of employees may also reflect increased competition in the pharmaceutical market globally.

Many of the original product companies also reported a decrease in the amount of prescription medicine marketing after GS. Original product companies often decrease their promotional activities (e.g. the marketing of product) when the generic competition begins [36]. In an earlier study, reduction in original product companies' marketing was one of the most commonly reported mechanism compensate for increased competition due to GS [7].

Generic product companies reported increased marketing activities directed to pharmacies in the early stage of GS. This is in line with a study conducted among Finnish pharmacists [37]. The finding is logical given that pharmacists play an important role when a patient decides whether or not to object to switching [17,38,39]. Marketing in the early stages following the introduction of GS may also provide a competitive advantage [40]. Previous Finnish studies have reported that GS has also changed the content of drug marketing directed to pharmacies, with a greater emphasis on price after GS [37,41].

Both the original and generic product companies reported difficulties in stock management, especially among the original product companies in the early stages of GS. Sales by the volume of generic pharmaceutical products have increased whereas sales by volume of original products have decreased after GS [42]. This may be one reason for some of the storage problems reported, such as overstocking by the original product companies. The market situation also changed more often after GS, because the price corridor is determined quarterly and prices of pharmaceutical products can be updated every two weeks. This also creates challenges for stock management. Previous studies have reported that stock management has also become more complicated in Danish [15] and Finnish [19] pharmacies as well as in Finnish wholesale pharmaceutical companies [20] due to GS.

Our findings did not strongly support the belief that GS would decrease the range of different packages (e.g. strength, size, pharmaceutical form) of pharmaceutical products for sale as it was anticipated before GS was introduced in Finland [27]. Furthermore, the results did not reveal that GS had impacted R&D in pharmaceutical companies. However, there were several companies that reported they decreased the number of packages for sale and the R&D of new pharmaceutical products in Finland. Accordingly, more detailed research on these topics are needed.

The study had some limitations. The survey questionnaires were posted to all companies that market substitutable medicines and had an office in Finland in 2004 and in 2007-2008. The response rate in both surveys was 56%. This is comparable or higher than in some survey studies posted to pharmaceutical companies [43,44] or companies from different industries in Finland [43,45], in which response rate has varied between 24 and 52%. However, the study population was small and this should be taken into account when interpreting the results of this study. This meant that, the categories of generic product companies and original product companies also included companies that marketed both original and generic products. If the study companies had been categorized differently, the results might have been different in some activities and between groups. In addition, pharmaceutical companies that marketed only generic or original products were slightly underrepresented among the survey respondents. This means caution is needed when generalizing the results of this study.

It should also be noted that results of this study are based on self-reports from the key persons of pharmaceutical companies. Consequently, it is possible that different sources (e.g. the statistics of company, the opinions of key persons) have been used when answering the questions of the survey. There is also the possibility for over- or underestimation in self-reported responses [46]. The questionnaires were returned anonymously and thus, the validity and reliability of the answers could not be investigated. However, some of our findings are in accordance with other sources, which support the validity of the responses. In addition, the background characteristics of the original product and generic product companies that responded in the 2004 and 2008 surveys were very similar. Besides, the responses especially in the original product companies were similar in both surveys. On the other hand, there were also variations in the trend of answers especially in the generic product companies between surveys. This could result from the fact that different companies responded in 2004 and 2008. Another possible reason is that impact of GS changed during the four years between the surveys. However, tracing the impact of GS in the same companies between years was not possible. Changes in the reported impact of GS on activities of companies between years 2004 and 2008 should be interpreted with caution.

This study was limited to the experiences of pharmaceutical companies in Finland. The operating environment of pharmaceutical companies and the practices of GS vary between countries. This means caution is needed when comparing the results from other countries. For instance, there is much variation in the size and structure of the pharmaceutical markets and the number of pharmaceutical companies that operate in the market between countries [1,34,36]. GS by pharmacists is mandatory in Finland and in some other countries (e.g. Sweden, Germany, Denmark) as well as in some of the states in United States [1,47]. However, in other countries (e.g. France, Italy, Netherlands, Norway, Portugal, Australia, Japan) as well as in most of the states in United States GS is allowed, but is not obligated [1,47-49]. Besides, GS has significantly promoted competition between pharmaceutical products [11,50] and influenced the sales and gross margins of companies in Finland [7] like in some other countries (e.g. United States, Netherlands, Sweden, Denmark, Germany) [4-6,8,10]. However, in some countries (e.g. Italy, Spain) the impact of GS on the sales of products and competition has been smaller [5,51]. Accordingly, the impacts of GS on activities other than competition in the companies may also vary between these countries.

## Conclusions

The study suggests that GS impacted the activities of pharmaceutical companies that represent mainly original or generic pharmaceutical products in Finland. In addition, there were some differences, although not statistically significant, between the surveyed original and generic product companies regarding the self-reported impacts of GS. The number of employees and amount of prescription medicine marketing decreased in many of the original product companies after the introduction of GS. In addition, the original product companies reported problems related to the storage of pharmaceutical products shortly after the reform. In the generic product companies, the prescription representatives' visits to pharmacies increased at the beginning of GS. Furthermore, GS caused problems with the storage of pharmaceuticals one year and five years after the introduction. This study was based a small group of surveyed companies and had some limitations. Further investigations are needed in this field.

## Competing interests

The authors declare that they have no competing interests.

## Authors' contributions

All authors participated in designing the study and discussed the findings of the study. JT collected the data, conducted the data analysis and drafted the manuscript. MB, PK and RA revised the manuscript. All authors read and approved the final manuscript.

## Pre-publication history

The pre-publication history for this paper can be accessed here:

http://www.biomedcentral.com/1472-6904/10/15/prepub
